# Supplementary material for: Is there a need for change in cancer survivorship care? A qualitative exploration of survivor experiences and needs at the Sydney Cancer Survivorship Centre Clinic
Source: Support Care Cancer. 2023 Oct 18;31(11):642. doi: 10.1007/s00520-023-08102-w (PMC10584705; doi:10.1007/s00520-023-08102-w)
Supplement: Supplementary file 1 — Supplementary file1 (DOCX 172 KB) [file 520_2023_8102_MOESM1_ESM.docx]

Is there a need for change in cancer survivorship care? A qualitative exploration of survivor experiences and needs at the Sydney Cancer Survivorship Centre Clinic.

Liam Anthony Obeid^1^, Haryana M Dhillon^2^, Sim Y Tan^1,3,4^, Janette L Vardy^1,4^

Affiliations
^1^ Concord Clinical School, Faculty of Medicine and Health, The University of Sydney, Camperdown, NSW, Australia.

^2^ The University of Sydney, Faculty of Science, School of Psychology, Centre for Medical Psychology & Evidence-based Decision-making, Camperdown, NSW, Australia.

^3^ Nutrition and Dietetics Department, Concord Cancer Centre, Concord Repatriation General Hospital, Hospital Rd, Concord, NSW, Australia.

^4^ Sydney Cancer Survivorship Centre, Concord Cancer Centre, Concord Repatriation General Hospital, Hospital Rd, Concord, NSW, Australia.

Corresponding author:

Prof. Janette Vardy MD PhD

Concord Cancer Centre,

Concord Repatriation General Hospital

Hospital Rd

Concord, NSW, 2137

Australia

Phone:  +61 2 9767 5000

Fax:      +61 2 9767 7934

Email: [janette.vardy@sydney.edu.au](mailto:janette.vardy@sydney.edu.au)

Word Count: Abstract = 243, Body = 2973

Key words: Cancer Survivorship, Model of care, Qualitative research, patient experience, unmet needs.

ORCID of Authors

Liam A Obeid 0000-0002-7670-1669

Haryana M Dhillon 0000-0003-4039-5169

Cindy Sim Yee Tan 000-0002-8188-9395

Janette L Vardy 0000-0002-5739-5790

**Abstract**

*Purpose:* Effective cancer survivorship care is contingent on a comprehensive understanding and management of the dynamic needs of cancer survivors. The Sydney Cancer Survivorship Centre (SCSC) clinic established a holistic, multidisciplinary model of survivorship care. We aimed to explore survivors’ experiences and perceptions of the clinic, and to identify their unmet needs.

*Methods*: Semi-structured focus groups (FG) involving participants recruited from the SCSC clinic were conducted by an experienced facilitator and observer using a guide covering: survivor perceptions of first SCSC clinic visit, services accessed, ongoing unmet needs, and how needs changed over time. FG were audio-recorded and transcribed. Interpretive description using a Framework approach was undertaken and participant characteristics summarised descriptively.

*Results*: Eight FG were conducted involving a total of 26 participants (mean age: 60), most were female (n=20), born in Australia (n=14) with breast cancer diagnoses (n=16). Four overarching themes were identified: i) perceptions of the SCSC clinic; ii) patient-centred care; iii) adjustment to illness; and, iv) external supports and resources. Participants valued the centralisation of multidisciplinary survivorship care at the SCSC clinic, which helped their recovery. Mitigating ongoing treatment sequelae, reassurance of good-health, normalisation of survivorship experiences, and handling caregiver stress represent some needs identified.

*Conclusions*: The SCSC clinic offers holistic, specialised care and reassurance to cancer survivors. Adjustment to the survivorship journey, inter-survivor shared experiences, and management of physical treatment sequelae were perceived as important in their recovery. Managing survivor needs is integral to improving long-term survivorship care.

**Introduction**

Cancer survivors are defined as any individuals diagnosed with cancer, from the day of diagnosis until the end of life.[1] In Australia, one in two people will be diagnosed with cancer by age 85.[2] Cancer survival rates have been increasing due to increased screening, and improved treatments and supportive care.[3] In 2016 the five year survival rate for all cancers in Australia was 69%.[2]

Effective cancer survivorship care is contingent on a comprehensive understanding and management of cancer survivors’ needs. Much of the existing literature pertaining to survivor experiences has focused on the acute diagnostic and treatment phase – pre-survivorship.(1) In 2005, the Institute of Medicine (IOM) released a report highlighting the deficiencies of existing cancer survivorship care models and their failure to address the longer-term, ongoing needs of cancer survivors.[1] Identifying the disparities between health care professionals and survivors understanding of survivorship needs, the IOM advocated for a transition in focus from the acute to longer-term survivorship care involving a focal point for survivorship follow-up.

Survival varies with cancer type and individual factors, as does quality of life. Survivors are faced with physical,[4] psychological,[5] and social [3-5] challenges following cancer diagnosis and treatment.[3] Common survivor experiences include side effects such as sustained peripheral neuropathy,[4, 6, 7] cognitive deficits,[8] cancer-related fatigue, depression and anxiety,[5, 7, 8] culminating in survivors’ construction of their new normal.[6, 9] These difficulties may gain prominence after acute treatment, with residual treatment effects, temporal deterioration, and new, late treatment effects impacting survivors’ functional status and quality of life.[3, 6-8]

These challenges are compounded by limited knowledge regarding what survivorship entails.[10] Unknown permanence of treatment sequelae,[9] fears of cancer recurrence,[3, 5, 6, 9] and psychological impacts on family [5, 10, 11] present challenges for survivors. The dichotomous desire to regain autonomy [4, 12, 13] over their lives, whilst seeking reassurance [13, 14] of good-health, is synonymous with adjustment to the survivorship phase. Notably, some survivors report positive growth, including: re-prioritised self- and family-focus,[12] with inter-survivor story sharing [6] fostering improved psychological and physical wellbeing.[12]

To better address the needs of cancer survivors, in 2013 the Sydney Cancer Survivorship Centre (SCSC) was established using a unique multi-disciplinary model of care based on the biopsychosocial model.[15] The SCSC includes a number of services, including a survivor-specific clinic, gym, and cottage. The clinic team, comprising an accredited exercise physiologist, dietitian, clinical psychologist, clinical nurse consultants, medical oncologists, and haematologist holistically address known risk factors for cancer recurrence and chronic illnesses, aiming to improve length and quality of life for survivors.[16-18]

Prior to clinic appointments survivors complete patient reported outcome measures (PROMs), screening physical and mental health (PROMs detailed in Supplementary File 1); multidisciplinary team (MDT) members review PROM responses in preparation for consultation. At the clinic visit survivors are allocated their own consultation room with their partners/caregivers. Each MDT member individually consults with the survivor, making recommendations based on the individual’s needs and condition. These recommendations are added to the Survivorship Care Plan prepared by staff prior to the visit. At the request of the referring doctor approximately 40% of medical oncology survivors have their ongoing follow-up in the SCSC clinic, with subsequent visits involving review by a medical oncologist and clinical nurse consultant. Follow-up is generally three monthly for the first three years, then six monthly to five years. In the gym an accredited exercise physiologist assesses and provides individualised classes for survivors. The Survivorship cottage runs programs including arts, music therapy, medical qigong, yoga and pilates, for survivors across the full cancer trajectory.

While patient satisfaction questionnaires are collected, no other formal patient-centred evaluation of the SCSC clinic has occurred. It is critical the clinic be evaluated against its ability to meet survivor needs and expectations, and if gaps exist, address them with changed or additional processes and programs. We aimed to explore the experiences and perceptions of cancer survivors attending the SCSC Clinic, and to identify their unmet needs.

**Methods**

*Study Design*

We used a qualitative research design, with focus groups (FG) responding to a semi-structured interview guide. The data were analysed using interpretive description; a goal-oriented approach to qualitative data analysis. It is underpinned by the core elements of grounded theory and thematic analysis, but employs a purpose-driven approach to data in lieu of an unanchored exploratory lens.[19] Interpretive description’s value lies in the applicability of findings to clinical practice, offering purpose-specific knowledge to a predetermined field. For the purposes of this study, data about survivor perspectives and experiences with cancer and the SCSC Clinic is directly applicable to the SCSC. These findings will form the basis of practical, evidence-based recommendations for the SCSC Clinic. Ethical approval was provided by the Sydney Local Health District Human Research Ethics Committee – CRGH (file number HREC/14/CRGH/23). All participants provided written, informed consent.

*Participants*

All participants were English-speaking cancer survivors who had attended the SCSC Clinic at least once. They must have been medical oncology or haematology patients who had completed primary treatment (surgery, chemotherapy and/or radiotherapy) with curative intent, and without evidence of cancer recurrence.[16]

*Procedure*

Recruitment of participants occurred via electronic letters, printed posters, and individual contact with existing SCSC clinic patients. FG, involving between 3-8 participants, were set-up and led by an experienced qualitative researcher and facilitator (HD) and first author (LO). All FG were held in the SCSC cottage, Concord, NSW, Australia. The cottage provided a quiet setting, physically distanced from the Survivorship Clinic and chemotherapy day unit associated with survivors’ medical oncology treatment. Written informed consent and demographic data forms were completed before FG started. FG were semi-structured following a discussion guide (Supplementary file 3) and ran for 30-60 minutes. The guide covered: perceptions of first SCSC Clinic visit, SCSC services accessed, ongoing unmet needs, and change in needs over time. FG were digitally audio-recorded and supplemented by notes taken by LO. Audio-recordings were transcribed verbatim by LO. FG continued until thematic saturation was achieved; no new themes arose for two consecutive FG. No repeat interviews were carried out. Transcripts were not returned to participants for comment and/or correction.

*Data Analysis*

Participant demographics were summarised descriptively. FG were analysed through interpretive description using a Framework approach. The Framework approach, summarised in Table 1, involves simultaneous data collection and analysis; researchers LO and HD independently, then collaboratively coded transcripts to develop a matrix of themes (Supplementary File 2), which were reviewed with data from subsequent FGs.[20]

*Table 1. Description of the Framework Approach.[21]*

| Stage of Framework Approach | Researcher Action |
| --- | --- |
| Familiarisation of content | Independently read FG transcripts. |
| Establishing thematic frameworks | Independently coded the major themes from transcripts, then collaboratively discussed them to establish a common thematic framework that reflected the overarching aims of the study. |
| Indexing | Using the framework, transcripts were re-read and coded into categories encapsulating the core concepts discussed in the FG: (i) perceptions of SCSC Clinic, (ii) patient-centred care, (iii) adjustment to illness, (iv) external supports and resources. |
| Tabulating | Themes were tabulated into matrix format, which included sub-themes and descriptors. |
| Mapping & interpretation | Interpretive description applied to FG data within the thematic matrices, with inter-theme connections developed and refined. |

FG = Focus Groups; SCSC = Sydney Cancer Survivorship Centre

*Minimising Biases*

To enhance the validity of findings, the following steps were taken to minimise bias. LO created memos to summarise impressions before and after FG discussions. Verbal debriefing occurred between LO and HD directly after FG and during transcription review. The coding framework was developed iteratively, involving multiple researchers, and coding undertaken by multiple researchers. Verbatim quotations from participants were used to demonstrate relationship between themes and participant’s own words.

**Results**

We recruited 26 participants with a mean age 60 years (range: 44 – 82) and a mean number of four visits to SCSC clinic (range: 1-15, standard deviation: 4). Most were female (n=20, 77%), born in Australia (n=14, 54%) with breast cancer diagnoses (n=16, 62%). They participated in a total of eight FG. Patient demographics are summarised in Table 2.

*Table 2. Focus Group (FG) patient demographics.*

| Participant Identification (ID) | FG | Sex | Age | Country of Birth | Cancer Diagnosis | Total Number of Clinics Attended* |
| --- | --- | --- | --- | --- | --- | --- |
| 1 | 1 | F | 58 | Australia | Pancreatic | 6 |
| 2 | 1 | F | 75 | Australia | Breast | 1 |
| 3 | 1 | M | 59 | England | Colorectal | 1 |
| 4 | 2 | F | 60 | Italy | Breast | 1 |
| 5 | 2 | M | 72 | Lebanon | Colorectal | 8 |
| 6 | 2 | F | 51 | China | Breast | 1 |
| 7 | 3 | F | 51 | Australia | Breast | 1 |
| 8 | 3 | M | 49 | Australia | Colorectal | 2 |
| 9 | 3 | F | 49 | Macedonia | Breast | 1 |
| 10 | 4 | F | 50 | Vietnam | Breast | 2 |
| 11 | 4 | F | 62 | Australia | Breast | 1 |
| 12 | 4 | F | 62 | Vietnam | Colorectal | 7 |
| 13 | 4 | F | 71 | France | Breast | 1 |
| 14 | 4 | F | 60 | Fiji | Breast | 1 |
| 15 | 5 | F | 60 | Romania | Colorectal | 4 |
| 16 | 5 | F | 50 | Serbia | Breast | 1 |
| 17 | 5 | M | 74 | Australia | Colorectal | 7 |
| 18 | 6 | F | 50 | Australia | Breast | 1 |
| 19 | 6 | F | 82 | Australia | Breast | 1 |
| 20 | 6 | F | 52 | Australia | Breast | 1 |
| 21 | 7 | F | 63 | Australia | Breast | 14 |
| 22 | 7 | M | 59 | Australia | Colorectal | 5 |
| 23 | 7 | M | 69 | Australia | Colorectal | 15 |
| 24 | 8 | F | 44 | Australia | Colorectal | 5 |
| 25 | 8 | F | 56 | Australia | Breast | 5 |
| 26 | 8 | F | 65 | Korea | Breast | 1 |

* Participants attending only 1 clinic visit had completed adjuvant chemotherapy within the past 6 months.

We identified four major themes from FG: (i) perceptions of the SCSC clinic; (ii) patient-centred care; (iii) adjustment to illness; and, (iv) external supports and resources. Themes are summarised in Table 3. Each theme is described below with key quotations summarised in Table 4 and in-text quotations accompanied by participant identification numbers.

*Table 3. Focus Group Themes for Participant Perspectives of SCSC Clinic and Survivorship Needs.*

| Perceptions of SCSC Clinic | Patient-Centred Care | Adjustment to Illness | External Supports and Resources |
| --- | --- | --- | --- |
| - First Clinic visit - Clinical team (staff) - Social connection and inclusive understanding - Questionnaires - Clinic discovery | - Individualised care - Quality of care - Reassurance - Technological assistance - Navigation of services | - Processing of cancer and changing mindset - Short-term and long-term needs | - Role of the caregiver - Role of the General Practitioner - Survivorship services |

*SCSC = Sydney Cancer Survivorship Centre*

*Theme 1. Perceptions of SCSC Clinic.*

Participants were unsure what to expect at their first SCSC clinic visit, recounting feeling overwhelming *“bombardment”* (P18) of information. The optimal time to move to the SCSC clinic after treatment varied, with some indicating feeling unable to engage with SCSC staff soon after treatment. Others felt prioritised within the patient-centred structure that provided time and privacy to interact with each MDT member. However, participants expressed disappointment with the unclear conclusion to the initial clinic visit. The clinic’s proximity to the chemotherapy suite was undesirable given the negative associations many participants developed during their treatment.

Participants expressed overwhelming praise and gratitude to the multidisciplinary clinic team. They appreciated the *“family”* (P19) atmosphere from staff who *“understand you”* (P14) and ask *“all the right questions”* (P13), whilst alluding to the value of deeper *“intrinsic bonds”* (P23) formed with other survivors. Participants particularly valued the exercise physiologist’s expertise, noting the importance of advice on physical capacity by *“someone knowledgeable about the surgery”* (P22). Seeing the psychologist at the initial clinic visit was more controversial, with some participants unprepared to face their psychological concerns at that time. Despite this participants acknowledged the importance of meeting the psychologist in facilitating future contact.

Participant evaluations of PROMs completed as part of follow-up were divergent. Some felt the PROMs were repetitive, *“endless”* (P22) and questioned *“what the purpose was”* (P20). Others valued the forced insight; *“it made you aware”* (P19) about aspects of survivorship that would otherwise have remained unconsidered, enabling survivors to monitor their progress. Participants recalled multiple pathways to learning about the SCSC clinic, including: medical oncologists, clinical nurse consultants, other SCSC services, and community programs; but most were referred by their medical oncologists.

*Theme 2. Patient-Centred Care*

The diversity of patient experiences at the clinic highlighted the importance of individualised, patient-centred care. Participants expressed a desire for greater control over frequency of follow-up, the clinic staff they saw, and greater flexibility of SCSC services for working survivors. Specifically, the concern about follow-up moving from three to six months was underpinned by the desire for regular reassurance; being able to express their health concerns and gain *“peace of mind”* (P10) in the *“safety blanket”* (P20) of the clinic. Participants commended clinic recommendations as individually tailored survivorship care plans that were specialised and superior to standard follow-up care. The need for greater use of technology to ease the travel burden for patients and to increase the efficiency of survivor-healthcare professional interactions were identified.

*Theme 3: Adjustment to Illness*

Participants unanimously agreed moving into the survivorship phase (after primary cancer treatment was completed) necessitated a change in expectations of recovery, life priorities, and management of existential threats. Lasting effects of treatment permeated all FG, with physical sequelae (e.g. chemotherapy-related side-effects), anxiety about present and future function, and uncertainty about mortality worrying all participants. Adjustment to survivorship was associated with variable symptoms and time-frames, with more persistent symptoms and longer-time frames manifesting as frustration with SCSC clinicians.

Much of the value in inter-survivor connections lay in shared stories of survivorship. These provided a barometer for recovery trajectory, normalising and validating survivor difficulties and experiences. Participants articulated a personal *“priority shift”* (P12) subsequent to threatened mortality, favouring self-fulfilment and self-actualisation over financial or material gains.

*Theme 4: External Supports and Resources*

Caregivers and partners were perceived to have contrasting impacts on participants. Within the clinic, they supplemented survivor experiences and recall of content by note-taking and reminding survivors of questions, enabling more authentic survivor engagement with clinic staff. Conversely, participants were acutely aware the burden their health had on their caregivers’ stress, feeling obliged to remain positive for their sake: presenting the caregiver conundrum.

General practitioner (GP) contributions to survivorship were inconsistent. Participants noted a lack of specialised oncological knowledge hindering GP’s capacity to help survivors. Breakdown in the continuity of care between hospital and GP care was a particular concern. While others appreciated the time, trust and GP’s superior knowledge of an individuals’ context.

Participants judged SCSC services associated with the clinic as extremely valuable in fostering inter-survivor relationships (through the cottage programs) and for receiving specialised care (e.g. in the gym). Greater support for these domains of survivorship care was considered important.

*Table 4. Key Participant Quotations for Major Coded Themes*

| Coded Subthemes | Participant Quotations |
| --- | --- |
| Theme 1 – Perceptions of the SCSC Clinic | |
| First Clinic Visit   - - - Clinic Experience     - Clinic Structure     - Clinic Location | *“It was too early … they wanted to help me and reach out but I wasn’t ready for it … I just wanted to heal and have peace”* (P18)  *“It was a bit of an information overload … I was a bit overwhelmed”* (P9)  *“I was the centre of attention, and it wasn’t about me imposing on them, but it was them seeing how they could look after me”* (P23)  *“I’d seen everybody, but everyone was gone, which was the disappointing thing. There was no one to sort of finish off that whole, tie that whole process together”* (P8)  *“I was just having flashbacks, and unless I had to be in that space [proximity to chemo-suite] … I don’t want to associate”* (P20) |
| Clinical Team (Staff) | *“I was just so impressed because they made it so easy. That was my first experience of having everything under the one roof, and having all these people available to me. It was so supportive; it was just amazing”* (P21)  *“There is no way I would’ve been as well as I am if I hadn’t seen the dietitian and exercise physiologist”* (P20) |
| Social Connection and Inclusive Understanding | *“it's only someone who's been through it really understands you; even the closest family members, unless they've been through it, do they really know what you're going through?”* (P05)  *“We don’t talk about cancer really … there are things that we don’t need to say because we understand each other, whereas out there it’s not quite the same”* (P21) |
| Patient Reported Outcome Measures | *“Do these forms have to be filled out so regularly? … I feel like, oh my goodness, these forms again!”* (P25)  *“You need to know that … that all of those things have been addressed”* (P23) |
| Clinic Discovery | *“[Medical oncologist] suggested that I come to the Survivorship Clinic”* (P9)  *“I went through the Concord Cancer Centre, so part of the procedures”* (P11) |
| Theme 2 – Patient-Centred Care | |
| Individualised Care   - - - Regularity of Follow-Up     - Fear of Shaming     - Control     - Timing of Services     - Accessibility | *“The meetings every 3 months are very important … if it’s too far, then you get complacent … but if it’s every 3 months, it keeps you vigilant”* (P5)  *“I feel like I’m getting into trouble. I feel like I’m getting schooled” (*P24)  *“I find the one thing is my independence … I’m used to being in control. Even now, I sort of like to be involved”* (P19)  *“There's kind of so much information coming, for me at least, in that short space of time, 6 months, there was so many things to do – there were side effects I was trying to understand, and who was going to look after me – I was kind of in a little bit of a blur”* (P16)  *"that was a big, confronting thing - to think that I might need a psychologist"* (P01)  *“As far as what is on offer, it is basically for retirees … I’m sorry, just because you’ve had cancer, or you have got cancer, doesn’t mean you don’t work”* (P1)  *"It's more what's available, and you're not totally cut off … the fact that I know they exist, and I have met the people, and I could just ring them up and start off if they want me to is good"* (P11) |
| Quality of Care | *“It does the job that I need … I don’t see it as being deficient in any way”* (P5)  *“Holistic approach to a person after they’d had treatment, and support, and learning to take care of ourselves … I was very impressed with the team of people”* (P13) |
| Reassurance | *“If there’s anything going on, you’re going to know about it … it’s reassuring because sometimes you just sit there in a quiet moment and wonder if you’re ok”* (P5)  *“It was more that reassurance … I just needed someone to say to me ‘you're ok, your body has recovered now’”* (P9) |
| Technological Assistance | *“Why don’t you organise Skype discussions? Or tele-conferencing? … and if it transpires that you have issues that need to be handled personally, then you make a visit personally”* (P15) |
| Navigation of Services | *“I had to wrap my head around what things were about”* (P16) |
| Theme 3 – Adjustment to Illness | |
| Processing of Cancer and Changing Mindset | *“They sort of say to you ‘you’re going to go back to life as normal’, but that's just not true, and I wish they wouldn’t say that – I found it really annoying … it’s just not true”* (P11)  *“I’ve changed the way I think in my head, the way I think about life, about money; I didn’t care about any of this. All I want is peace and my health”* (P20)  *“Having your mortality threatened is such a big mental thing … takes away your sense of certainty … it takes a fair bit of getting your head ‘round all that”* (P23) |
| Short-term and Long-term Needs | *“In my mind it’s not as sharp … like should I be at work, should I be driving a car? Double questioning myself, double doubting myself all the time”* (P18)  *“My fingers and feet. It’s not just numb, it’s very painful … I got very depressed. I couldn’t sleep or eat … every medicine had a side effect; can’t sleep, constipation, nausea … I wanted to die inside but I couldn’t tell anyone”* (P26)  *“It would be good to talk about other things in our life … questions to cover that sort of permanency, and I mention it because, I thought ‘how do I know [if] it’s relevant or not?’”* (P15)  *“You’ve got to think about performance at work, and at home, with family … it’s forever. It’s, ‘yes, we’ve survived … for now’”* (P18) |
| Theme 4 – External Supports and Resources | |
| Role of the Caregiver | *“My daughter came with me too … so she took notes, and she reminded me … of different things… asked questions … I was just focused on what they were saying and weren’t worried about writing it down”* (P19)  *“The biggest thing in my case like my wife, my children, my grandchildren, if they see me sitting down moping and feeling sorry for myself, then they’re going to get down in the dumps, and that would really worry me. Then I would feel I’m letting them down, and they would get depressed as well”* (P5)  *“[Psychologically] it was very hard. But what made that harder was that I was worried that [my wife] was worried; it was affecting me because I knew she was being strong for me … it just added another layer of psychological stress”* (P23) |
| Role of the GP | *“If you have a good GP, it’s so important … [a] very good support thing to see my GP because I didn’t have the support group … I can talk to [him] because I’ve known him for so long”* (P13)  *“I feel like [communication between GPs and hospital staff was] not seamless. I feel like there are so many gaps, that we’re falling in between those gaps”* (P16) |
| Survivorship Services | *“I [would] like to have the gym continued, to continue with it, because I finished with the gym and can’t go there anymore – they only give you a certain period of time”* (P14) |

**Discussion**

We analysed cancer survivor perspectives on the SCSC Clinic and explored their unmet needs. Overall, participants were supportive and appreciative of the SCSC clinic and associated services. They valued the patient-centred, multidisciplinary approach to care that enabled them to navigate the physical and psychosocial issues encountered during survivorship.

Our findings confirm the clinic addresses the IOM’s recommendation for centralised, multidisciplinary approaches to long-term survivorship care.[1] Despite initial concerns about expectations and location, the SCSC Clinic has successfully provided survivors with a specialised focal point for survivorship follow-up, circumventing the extant fragmented system. Survivors particularly valued the high-quality, holistic care received, addressing physical, psychological, and social limitations of survivors. This contrasts with their perceptions of the generic healthcare received in the community, illustrating the value of specialised care in this high need population.

Consistent with other studies, survivors valued the clinic for the reassurance it provided about their health status.[13, 14] However, it has been suggested the emphasis on in-person follow-up misleads survivors about optimal disease monitoring (i.e. not necessarily physical examination), and may increase anxiety and survivor dependence on healthcare professionals.[22] Dependence on healthcare professionals may reduce self-efficacy for survivors to engage in self-management of their health.[4, 12, 13] Balancing these conflicting needs presents a complex problem requiring further investigation.

Our results support existing reports of long-term needs of cancer survivors. Our population reported persistent neurological, cognitive, and psychological impacts of treatment into survivorship, corroborating Burg,[4] Tanay,[7] and Harrington’s [8] work regarding sustained treatment sequelae. Predictably, this impacted survivors’ quality of life and functional capabilities.[3, 7] Frustration with clinicians dismissing ongoing treatment-induced effects echo the findings of Tanay.[7] Recognising the value of acknowledging symptoms and acknowledging the limitations of what can be done to intervene, may reduce this frustration.

The psychological impacts of cancer diagnosis and survivorship are well established.[3, 6, 12] The reticence to talk with a clinical psychologist at the first clinic visit may have been due to participants early stage of survivorship and ongoing adjustment to illness. Although for some, introduction to clinical psychologists during the first visit facilitated later contact, at a time survivors felt ready to address their psychological concerns.

Fear of cancer recurrence, reprioritised life values, and secondary cancer impacts on family were identified, and reflect and add to the wider literature.[5, 11, 12, 14] We explored the survivor-caregiver dynamic in greater detail than previous work. Our results indicate a bi-directional [5, 11] impact of survivor and caregiver stress, suggesting the need for healthcare professionals to attend to both survivor and caregiver psychological wellbeing and the complex interplay between them.

Our findings extend the work of McCaughan [6] and Connerty & Knott,[12] providing further explanation for survivor interest in support groups. Beyond the increased understanding and normalisation of survivorship experiences, inter-survivor relationships and shared experiences offered a reference point for recovery trajectory and were a source of genuine empathy. This validated survivors’ experiences beyond what non-survivor family and friends could provide.

GP involvement was highlighted as a positive, with patients wanting involvement of GP’s who knew them well, but they also expressed frustration with GP’s limited cancer knowledge. Better quality and more frequent communication between specialists and GPs, and improved GP cancer education were emphasised. Use of technology, specifically tele-health, was raised as a means of reducing travel burdens for survivors in follow-up care, whilst maintaining the reassurance of regular monitoring. Integrally, tele-conferencing may indirectly enhance patient autonomy by encouraging patient education for physical signs and symptoms of disease recurrence.[13] Interestingly, in response to the COVID-19 pandemic, the SCSC clinics rapidly transitioned to telehealth consultations in March 2020, incorporating electronic PROM collection to facilitate MDT member knowledge of survivor symptoms. This transition demonstrates the feasibility and acceptability of telehealth in this setting and presents a viable, long-term initiative for change.

*Implications & Recommendations*

Exploring survivor experiences and needs at the SCSC Clinic is integral to continuous quality improvement in delivery of long-term survivorship care. Our results confirmed and augmented existing survivorship literature, and have been synthesised into recommendations to improve SCSC clinic care outlined in Table 5. The recommendations have been categorised as those able to be implemented in the short- or long-term, as well as by the projected resources required to implement each (low versus high). High resource recommendations are those requiring systems change, longer planning, and more time to set-up and implement.

*Table 5. Recommendations for Sydney Cancer Survivorship Centre clinic improvements.*

| Time Frame | Resources required for implementation | Recommendations |
| --- | --- | --- |
| Short Term | Low | - Provide information to survivors about what to expect during the first clinic; e.g., provide an A4 summary sheet and/or short video of how the Clinic runs. - Survivors should be advised to bring a friend or family member (i.e. caregiver) with them to clinics. - An individual (e.g. final team member seeing a survivor or the Survivorship Nurse Consultant) should: (i) verbally inform survivors they have seen every team member and their clinic review is complete; (ii) provide the follow-up plan, including recommended dates; and, (iii) answer remaining questions. - Communicate to survivors the purpose and value of discussing symptoms (i.e. acknowledgement is important even if treatment not available). - Better clinician communication of interventions available to mitigate effects of long-term treatment sequelae (e.g. peripheral neuropathy). - Use of patient connect services to link people recently completed treatment to longer-term survivors for support. - Educate survivors and caregivers regarding the interaction between each of their feelings of stress. - Advise caregivers to be aware of their own mental health and wellbeing, and to seek professional help if required. |
|  | High | - Provide annual feedback to survivors and GPs on their PROMs. - Feedback should include individual progress, change in symptoms, mood and health. - Access to stories from other survivors with similar diagnosis, age, and sex about comparative recovery trajectories (e.g., video interview, etc). - Make it clear to survivors that, based on needs assessed in follow-up clinics, access to additional MDT members beyond the medical oncologist and clinical nurse consultant is available. |
| Long Term | Low | - Telehealth (i.e. video) consultations for longer-term clinic follow-up based on survivor preference, symptom profile, and needs. - Introduction should follow a period of in-person clinic reviews, during which survivor education and confidence regarding symptom recognition and self-examination are developed. - Improve communication between the SCSC clinic and GPs of survivors about individuals’ health, progress, and plans. |
|  | High | - Alternate locations for the SCSC clinic to distinguish it from chemotherapy treatment. - Increased allocation of resources to the adjunctive SCSC clinic services, including the gym and cottage. - Increase total capacity of each service to accommodate a greater number of survivors, length of time survivors may access the service, and increase service hours to accommodate working survivors. - Greater community education about cancer-specific needs and capabilities, specifically for GPs and personal trainers at community gyms, should be supported with information seminars run by the SCSC clinic MDT members. |

*Note: classification of low and high resource recommendations was based on assumptions regarding resources (staff, Information Technology, policy change, etc) required to implement them in practice.*

*Limitations*

This qualitative study was limited by participant demographics which favoured female, breast cancer survivors born in Australia, and is particularly pertinent given the diversity in survivor needs relative to their cancer diagnosis.[4] A further limitation is the self-selection bias in FG participation. It is possible the cohort of participants did not reflect the opinions of the majority of survivors, rather representing the extremes of opinion, both good and bad, about the SCSC clinic. The majority of the participants had experienced the initial clinic only and their views may differ from those receiving longer-term follow-up in the SCSC clinic. Participant responses included their experiences during the active treatment period and other SCSC services (e.g. Cottage activities, gym) and were not exclusive to the SCSC clinic. Whilst difficult to measure, the engagement of an experienced facilitator, use of semi-structured focus group discussion guide, adherence to an iterative methods and self-reflection on biases through memoing pre- and post-data analysis were integral to mitigating biases, thus improving the accuracy, reliability and generalisability of findings.

*Future Directions*

Future research should seek to recruit a sample of survivors more reflective of the tumour groups attending SCSC Clinic and those who have attended SCSC clinic for longer follow-up periods to observe temporal changes in needs and explore their congruence with the SCSC clinic’s MDT. Other research questions include the exploration of diagnosis-specific survivor needs, and the relevance of this clinic model to adult survivors of paediatric cancer.

*Conclusions*

Our dual aims were to explore cancer survivor experiences and perceptions of the SCSC clinic, and identify their unmet needs. Positive participant experiences indicate the success of the SCSC clinic in centralising and delivering holistic, long-term survivorship care. Recommended improvements address survivor concerns about the clinic and their unmet needs, and include ensuring continuity of care with GPs, and managing survivor expectations of the Clinic. Participants value inter-survivor shared experience and recognise bidirectional survivor-caregiver stress. This model of care provides an approach able to achieve the IOM recommendations regarding provision of efficient long-term survivorship care. **Declarations**

*Ethics approval and consent to participate:* This study was approved by the Sydney Local Health District Human Research Ethics Committee – CRGH (file number: HREC/14/CRGH/23) on 3^rd^ July, 2018. All participants provided written, informed consent to participate in the study in accordance with the Australian National statement on ethical conduct in human research (2018). The participant consent form explicitly outlined participants freely agreed to participate in the study, having been made aware of the potential benefits and risks of participation, and the potential outcomes of the data provided during the study.

*Consent for publication:* All participants provided written, informed consent regarding the potential publication of this report. The information sheet they were provided with as part of the consenting process outlined that “a report of this study may be submitted for publication”. This possibility was also addressed during recruitment and verbal consenting of participants.

*Competing interests:* Liam Obeid did not have active or direct associations with the Concord Cancer Centre or Sydney Cancer Survivorship Centre. JV is Director of the SCSC, ST provides dietitian services for SCSC. JV, HD, ST were involved in the design and set-up of the SCSC and model of care. HD declares receiving honoraria paid to her institution from BMS, Janssen-Cilag, and MSD.

*Authors’ contributions:* Liam Obeid (MD, BSc(Adv.)) was a medical student at the time of this study. He recruited participants, organised FG and assisted the running of FG. FG were facilitated by Haryana Dhillon (B.Sc MA(psych) PhD; female with >15 years experience in qualitative and mixed methods research). Data was coded together by Liam Obeid and Haryana Dhillon. Janette Vardy (BMed (Hons) FRACP PhD; female Medical Oncologist with >20 years experience in cancer clinical research) and Sim Y Tan (BAppSci, MNDiet, PhD); female dietitian with >10 years experience as an Oncology Specialist Dietitian) oversaw the completion of this research as the expert advisor and research tutor, respectively.

*Funding:* No funding supported this study. Prof Vardy is funded by an NHMRC Investigator Grant and ST holds a post-doctoral position supported by the same grant [Number: APP1176221]. Prof Vardy was previously supported by a Practitioner Fellowship from the National Breast Cancer Foundation, Australia.

*Availability of data and material:* Audio-recordings and verbatim transcripts of focus groups have been retained to enable data transparency; these materials are available for review on request. The thematic code matrix upon which this report is based can be found in Appendix 1.

**Acknowledgements:**

We would like to thank all the participants for generously donating their time to take part in the study. Thank you also to Kain Phansuwon and Yu Chaing (William) Lai who assisted with the recruitment of study participants.

**References**

1. Hewitt, M., S. Greenfield, and E. Stovall, *From cancer patient to cancer survivor: lost in transition - an American Society of Clinical Oncology and Institute of Medicine Symposium*. 2005, Washington, DC: National Academies Press.

2. Welfare, A.I.o.H.a., *Cancer in Australia: In Brief 2019*, A. Government, Editor. 2019, AIHW: Canberra.

3. Griffith, K.A., D.B. McGuire, and M. Russo, *Meeting Survivors’ Unmet Needs: An Integrated Framework for Survivor and Palliative Care.* Seminars in Oncology Nursing, 2010. **26**(4): p. 231-242.

4. Burg, M.A., et al., *Current unmet needs of cancer survivors: analysis of open-ended responses to the American Cancer Society Study of Cancer Survivors II.* Cancer, 2015. **121**(4): p. 623-30.

5. Harrison, J.D., et al., *What are the unmet supportive care needs of people with cancer? A systematic review.* Support Care Cancer, 2009. **17**(8): p. 1117-28.

6. McCaughan, E., et al., *Exploring and comparing the experience and coping behaviour of men and women with colorectal cancer after chemotherapy treatment: a qualitative longitudinal study.* Psychooncology, 2012. **21**(1): p. 64-71.

7. Tanay, M.A.L., J. Armes, and E. Ream, *The experience of chemotherapy-induced peripheral neuropathy in adult cancer patients: a qualitative thematic synthesis.* Eur J Cancer Care (Engl), 2017. **26**(5).

8. Harrington, C.B., et al., *It's not over when it's over: long-term symptoms in cancer survivors--a systematic review.* Int J Psychiatry Med, 2010. **40**(2): p. 163-81.

9. Ratcliff, C., et al., *Examining cancer survivorship trajectories: Exploring the intersection between qualitative illness narratives and quantitative screening instruments.* Palliat Support Care, 2018. **16**(6): p. 712-718.

10. Bernat, J.K., et al., *Symptom burden and information needs in prostate cancer survivors: a case for tailored long-term survivorship care.* BJU Int, 2016. **118**(3): p. 372-8.

11. Ferrell, B.R., et al., *Family Caregivers: A Qualitative Study to Better Understand the Quality-of-Life Concerns and Needs of This Population.* Clin J Oncol Nurs, 2018. **22**(3): p. 286-294.

12. Connerty, T.J. and V. Knott, *Promoting positive change in the face of adversity: experiences of cancer and post-traumatic growth.* Eur J Cancer Care (Engl), 2013. **22**(3): p. 334-44.

13. Cox, A., et al., *Cancer Survivors' Experience With Telehealth: A Systematic Review and Thematic Synthesis.* J Med Internet Res, 2017. **19**(1): p. e11.

14. Bender, J.L., et al., *Thyroid cancer survivors' perceptions of survivorship care follow-up options: a cross-sectional, mixed-methods survey.* Support Care Cancer, 2016. **24**(5): p. 2007-2015.

15. Engel, G.L., *The need for a new medical model: A challenge for biomedicine.* Science, 1977. **196**: p. 129–136.

16. Vardy, J.L., et al., *Health status and needs of cancer survivors attending the Sydney Survivorship Centre clinics and programmes: a protocol for longitudinal evaluation of the centre's services.* BMJ Open, 2017. **7**(5): p. e014803.

17. Tan, S.Y., et al., *Health concerns of cancer survivors after primary anti-cancer treatment.* Support Care Cancer, 2019. **27**(10): p. 3739-3747.

18. Vardy, J.L., et al., *What happens to cancer survivors attending a structured cancer survivorship clinic? Symptoms, quality of life and lifestyle changes over the first year at the Sydney Cancer Survivorship Centre clinic.* Support Care Cancer, 2021. **29**(3): p. 1337-1345.

19. Thorne, S.E., *Interpretive Description: Qualitative Research for Applied Practice*. Second ed. 2016, 711 Third Avenue, New York, NY: Routledge; Taylor & Francis.

20. Gale, N.K., et al., *Using the framework method for the analysis of qualitative data in multi-disciplinary health research.* BMC Medical Research Methodology, 2013. **13**(1): p. 117.

21. Muscat, D.M., et al., *Qualitative insights into the experience of teaching shared decision making within adult education health literacy programmes for lower-literacy learners.* Health Expect, 2017. **20**(6): p. 1393-1400.

22. Beaver, K. and K.A. Luker, *Follow-up in breast cancer clinics: reassuring for patients rather than detecting recurrence.* Psychooncology, 2005. **14**(2): p. 94-101.

**Supplementary materials**

*Appendix 1 – Patient-reported outcome measures administered in Sydney Cancer Survivorship Clinic*

| **Outcome** | **Measure** |
| --- | --- |
| Distress | Distress Thermometer |
| Symptoms | Patient’s Disease and Treatment Assessment Form |
| Physical activity | modified Godin Leisure Time Exercise Questionnaire LTEQ |
| Diet | 3-day or 24-hour recall food diary  Food questionnaire |
| Health-related quality of life questionnaire | Functional Assessment of Cancer Therapy – General (FACT-G) |
| Disease-specific health related quality of life modules | Functional Assessment of Cancer Therapy -modules:   - breast - gastric - oesophageal - liver (biliary, pancreatic), - lung, - gynaecology (ovarian or endometrial) |

*Appendix 2 – Coded Matrix of Themes*

| **Theme** | **Sub-Theme** | **Descriptor** |
| --- | --- | --- |
| 1. Perceptions of SCSC Clinic | 1.1. 1^st^ Clinic Visit   - 1.1.1. Experience of Clinic (preparation, timing, intensity) - 1.1.2. Structure of Clinic - 1.1.3. Location of Clinic | - How prepared survivors felt – i.e. if they knew what to expect - Timing of first clinic in relation to oncological treatments - Whether survivors felt overwhelmed by the quantity and intensity of the advice/recommendations - Comments on the clinic structure; being seen in their own room, with clinic staff entering and exiting - The location of the SCSC clinic, for example, its proximity to the chemotherapy ward |
|  | 1.2. Clinical Team (Staff) | - Members of the clinic team that were or were not seen as helpful - Clinic recommendations that were or were not deemed valuable - Members of the clinic team that were deemed in/appropriate at different stages of survivorship |
|  | 1.3. Social Connection / Inclusive Understanding | - Understanding that other people had been through the same cancer and treatment they had. - Appreciating the SCSC clinic team for their continued emotional support and investment in survivors and their progress - Other cancer survivors- understanding the past and present struggles of other survivors without it needing to be discussed |
|  | 1.4. Patient reported outcome measures (PROMs) | - Comments on PROMs as being too long, annoying or repetitive, or useful tools in tracking progress and reflection |
|  | 1.5. Clinic Discovery | - Various routes for hearing about clinic, including from treating oncologist, word-of-mouth endorsement by other patients, advertising, or external (to Concord Hospital) health care professionals |
| 2. Patient-Centered Care | 2.1. Individualised Care   - 2.1.1. Regularity of follow up - 2.1.2. Fear of Shaming - 2.1.3. Consistency in Clinician - 2.1.4. Control - 2.1.5. Timing of Services - 2.1.6. Accessibility | - Comments on whether repeat clinic appointments were adequately timed, or needed to be more or less regular - Fear of clinic team shaming patients if they did not adhere to their recommendations - Having more control over which clinic team members patients saw, according to their specific needs at that time - Comments on when to start the clinic in the survivorship journey - Comments on the convenience of clinic hours |
|  | 2.2. Quality of Care | - Patient satisfaction or dissatisfaction of care provided at the clinic with respect to the breadth of staff available and the teamwork between staff members |
|  | 2.3. Reassurance | - The comfort of having good-health confirmed in clinics, and of knowing a specialised team exists and remains available for help |
|  | 2.4. Technological Assistance | - The potential to use tele-conferences or video-calls to cater for individuals who live far distances, and to audio-record & transcribe clinic interactions for efficient note-taking |
|  | 2.5. Navigation | - Understanding SCSC clinic role supporting navigation of the healthcare system - Understanding SCSC clinic specific role with respect to other Survivorship services, and being able to enroll in any of them |
| 3. Adjustment to Illness | 3.1. Processing of Cancer / Mindset | - Extent to which patients thought they would recover e.g. fully - Changes in thinking and re-prioritisation following cancer - Difficulty in coming to terms with the prospect of mortality - The changing needs, or recognition of changing needs with time - Opinions on the terminology of ‘Survivorship’ |
|  | 3.2. Short and Long Term Needs | - Enduring physical effects of treatment, including surgery, chemotherapy, radiotherapy or hormone replacement therapy - The way in which these ongoing issues impeded on survivors’ ability to interact and involve themselves with the SCSC services - Understanding their experience is normal for a cancer survivor - Lasting effects of treatment on self-image - When SCSC clinic team asks questions about patient condition for which they don’t have a solution to the problem - When SCSC clinic staff do not meet all the needs of the survivor |
| 4. External Supports and Resources | 4.1. Role of the Caregiver | - When individuals diagnosed with cancer have to help emotionally support their partners/spouses, e.g. by staying strong - Caregivers taking notes during the clinic to enable survivors to engage more directly with the services and clinic team |
|  | 4.2. Role of the General Practitioner (GP) | - If GPs have the knowledge to adequately identify and manage oncological re-occurrence or complications - Desire for better or more frequent interactions between primary care practitioners and the treating team in hospital - Greater understanding between GPs and hospital doctors |
|  | 4.3. Survivorship Services | - Whether the resources funding cottage activities are sufficient - Difficulties with gym attendance in terms of finite time survivors are allowed to go to the gym, but also how many people are able to be in the classes at the same time - Desire for the general public to have greater understanding about what it means to be a cancer survivor and their limitations |

*Appendix 3 – Interview Guide for Focus Groups*

Qualitative substudy protocol (version 6 – 1^st^ May, 2018) – extract

Semi structured Focus Group and Interviews:

Interviews will be guided by a semi-structured interview schedule. Topics discussed will include but are not limited to:

i) What are your current cancer survivorship needs?

ii) Are these needs being met? If so where, and how. If not, why?

iii) How have your needs changed over your cancer journey

iv) How is the Sydney Cancer Survivorship Centre clinic meeting your needs? Is this good or bad? What could be done better?

v) What helps or hinders you from having your needs met?

*Appendix 4 – COREQ Checklist*

| **Item** | **Question/topic** | **Page number** |
| --- | --- | --- |
| **Domain 1: Research team and reflexivity** | | |
| **Personal Characteristics** | | |
| 1 | Interviewer/facilitator  Which author/s conducted the interview or focus group? | 9 |
| 2 | Credentials  What were the researcher’s credentials? E.g. PhD, MD | 5 (author contributions) |
| 3 | Occupation  What was their occupation at the time of the study? | 5 (author contributions) |
| 4 | Gender  Was the researcher male or female? | 5 (author contributions) |
| 5 | Experience and training  What experience or training did the researcher have? | 5 (author contributions) |
| **Relationship with participants** | | |
| 6 | Relationship established  Was a relationship established prior to study commencement? | Not reported |
| 7 | Participant knowledge of the interviewer  What did the participants know about the researcher? e.g. personal goals, reasons for doing the research | Not reported |
| 8 | Interviewer characteristics  What characteristics were reported about the interviewer/facilitator? e.g. Bias, assumptions, reasons and interests in the research topic | 5 (author contributions) |
| **Domain 2: study design** | | |
| **Theoretical framework** | | |
| 9 | Methodological orientation and Theory  What methodological orientation was stated to underpin the study? e.g. grounded theory, discourse analysis, ethnography, phenomenology, content analysis | 8 |
| **Participant selection** | | |
| 10 | Sampling  How were participants selected? e.g. purposive, convenience, consecutive, snowball | 8 |
| 11 | Method of approach  How were participants approached? e.g. face-to-face, telephone, mail, email | 9 |
| 12 | Sample size How many participants were in the study? | 9 |
| 13 | Non-participation How many people refused to participate or dropped out? Reasons? | Not reported |
| **Setting** | | |
| 14 | Setting of data collection Where was the data collected? e.g. home, clinic, workplace | 9 |
| 15 | Presence of non-participants Was anyone else present besides the participants and researchers? | Not reported |
| 16 | Description of sample What are the important characteristics of the sample? e.g. demographic data, date | Page 11-12, table 2 |
| **Data collection** | | |
| 17 | Interview guide Were questions, prompts, guides provided by the authors? Was it pilot tested? | Supp file 2. |
| 18 | Repeat interviews Were repeat interviews carried out? If yes, how many? | 9 |
| 19 | Audio/visual recording Did the research use audio or visual recording to collect the data | 9 |
| 20 | Field notes Were field notes made during and/or after the interview or focus group? | 9 |
| 21 | Duration What was the duration of the interviews or focus group? | 11 |
| 22 | Data saturation Was data saturation discussed? | Not reported |
| 23 | Transcripts returned Were transcripts returned to participants for comment and/or correction? | 11 |
| **Domain 3: analysis and findings** | | |
| **Data analysis** | | |
| 24 | Number of data coders How many data coders coded the data? | 9 |
| 25 | Description of the coding tree Did authors provide a description of the coding tree? | 9, supp file 1 |
| 26 | Derivation of themes Were themes identified in advance or derived from the data? | 9 |
| 27 | Software What software, if applicable, was used to manage the data? | 9 |
| 28 | Participant checking Did participants provide feedback on the findings? | 10 |
| **Reporting** | | |
| 29 | Quotations presented Were participant quotations presented to illustrate the themes / findings?  Was each quotation identified? e.g. participant number | Results  Pages 12-19 |
| 30 | Data and findings consistent Was there consistency between the data presented and the findings? | Results  Pages 12-19 |
| 31 | Clarity of major themes Were major themes clearly presented in the findings? | Page 12 |
| 32 | Clarity of minor themes Is there a description of diverse cases or discussion of minor themes? | Pages 13-19 |
